# Degradative and Non-Degradative Roles of Autophagy Proteins in Metabolism and Metabolic Diseases

**DOI:** 10.3389/fcell.2022.844481

**Published:** 2022-05-13

**Authors:** Kenta Kuramoto, Congcong He

**Affiliations:** Department of Cell and Developmental Biology, Feinberg School of Medicine, Northwestern University, Chicago, IL, United States

**Keywords:** autophagy-related gene, mitophagy, lipophagy, ER-phagy, adipose tissue, liver, beta cell (β cell), secretion

## Abstract

Autophagy is a stress-induced lysosomal degradation pathway regulated by evolutionarily conserved autophagy-related (ATG) genes. Recent research has revealed that autophagy plays an important role in the regulation of energy metabolism, development of metabolic tissues, and pathogenesis of metabolic disorders. Bulk and selective degradation by autophagy helps maintain protein homeostasis and physiological function of cells. Aside from classical degradative roles, ATG proteins also carry out non-classical secretory functions of metabolic tissues. In this review, we summarize recent progresses and unanswered questions on the mechanisms of autophagy and ATG proteins in metabolic regulation, with a focus on organelle and nutrient storage degradation, as well as vesicular and hormonal secretion. Such knowledge broadens our understanding on the cause, pathophysiology, and prevention of metabolic diseases including obesity and diabetes.

## Introduction on the Autophagy Pathway

**FIGURE 1:**
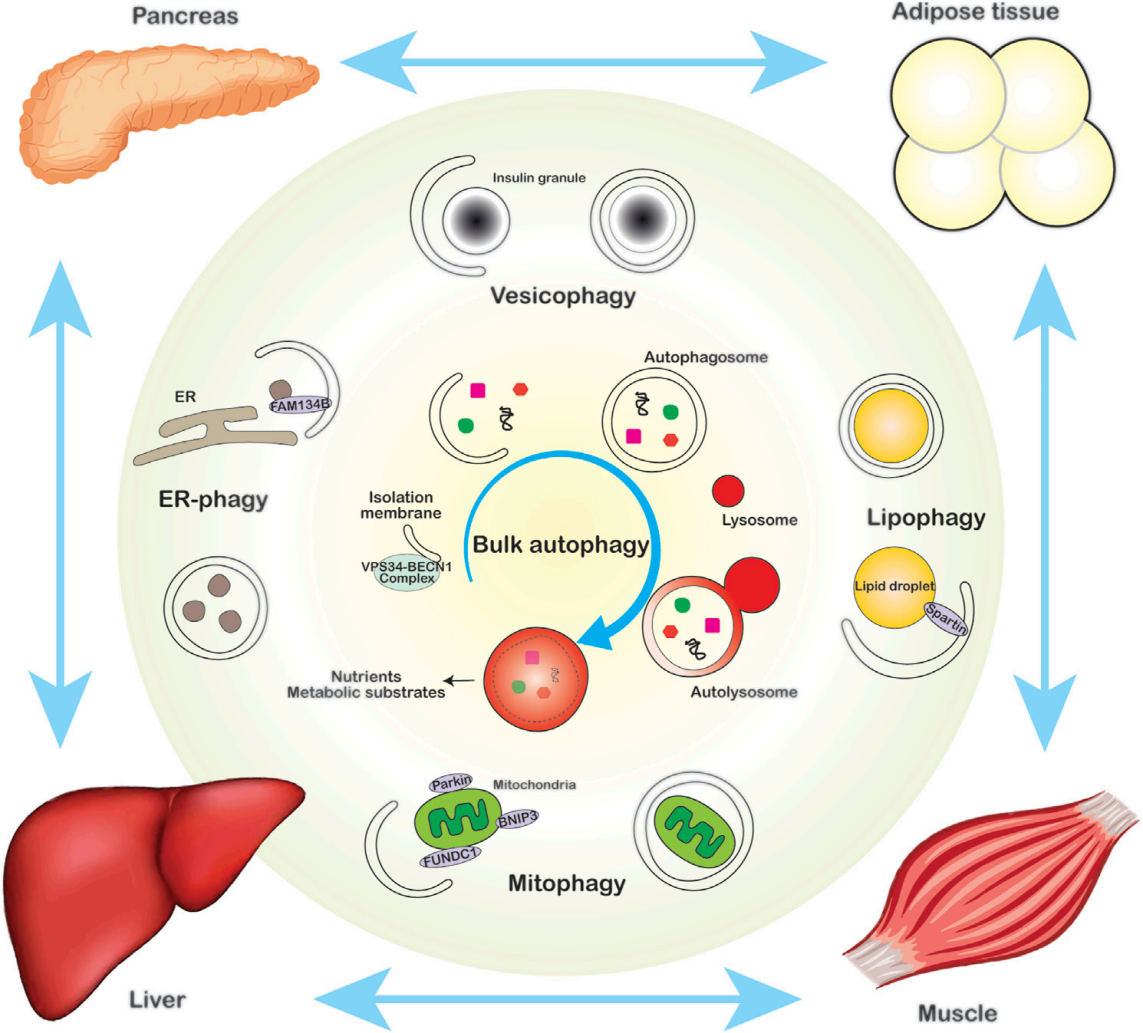
The role of autophagy in intracellular and systemic energy homeostasis. Bulk autophagy (center) and organelle-specific selective autophagy, such as vesicophagy (top), ER-phagy (left), lipophagy (right) and mitophagy (bottom), play pivotal roles in maintaining intracellular energy homeostasis via supplying nutrients and removing defective organelles. Autophagy also regulates systemic metabolic homeostasis via the cross-talk among metabolic organs in a non-cell autonomous manner.

Autophagy is an evolutionally conserved intracellular degradation system, which degrades various types of cellular components by delivering cargos into the lysosome that contains degradative enzymes (Dikic and Elazar, 2018; Aman et al., 2021). Various cellular stress stimuli, including nutrient depletion, exercise and oxidative stress, induce autophagy. Autophagy plays a critical role in both supplying metabolites and eliminating damaged cellular compartments to maintain cellular homeostasis. Dysregulated autophagy is associated with the pathogenesis of a variety of metabolic disorders, such as obesity and diabetes (Rocchi and He, 2015). Based on differences in the mechanism of lysosomal cargo delivery, autophagy is categorized into three major types, macroautophagy, microautophagy, and chaperone-mediated autophagy (CMA). Macroautophagy involves the formation of double-membrane autophagosomes and requires the coordination and execution of more than 40 autophagy-related (ATG) genes. Based on cargo selectivity, macroautophagy can be non-selective bulk degradation of the cytosol (bulk autophagy), or selective degradation of cargos via specific cargo receptors (selective autophagy). Depending on cargo types, selective autophagy can be further classified into several subtypes, such as lipophagy (autophagy of lipid droplets), mitophagy (autophagy of mitochondria), and ER-phagy (autophagy of the endoplasmic reticulum/ER) (Figure 1). In comparison, microautophagy degrades target components by directly engulfing cargos into the lysosome, through the invagination of the lysosomal membrane in an ESCRT (endosomal sorting complexes required for transport) machinery-dependent manner. CMA, on the other hand, is the transport of a specific subset of proteins via a lysosomal membrane receptor and transporter LAMP2A (lysosomal associated membrane protein-2 type A). The CMA substrates must possess a KFERQ-like pentapeptide sequence, which binds to heat shock-cognate chaperon 70 KDa (HSC70). The HSC70-substrate complex then interacts with LAMP2A, through which the target proteins are translocated into the lysosome. Based on existence of the predicted KFERQ-like targeting motif, approximately 30% of total cellular proteins are potentially degraded via CMA (Alfaro et al., 2018).

Macroautophagy is the main focus of this review. Various types of stress stimuli induce autophagy via multiple signaling cascades, including activation of AMP-activated protein kinase (AMPK) and inhibition of mammalian target of rapamycin (mTOR) signaling pathways. Both AMPK and mTOR can phosphorylate the autophagy-initiating Unc-51-like kinase 1 (ULK1/ATG1) complex, which is composed of ULK1/ATG1, ATG13, ATG101 and RB1-inducible coiled-coil protein 1 (RB1CC1/FIP200). While AMPK-mediated ULK1 phosphorylation activates ULK1 and autophagy, mTOR-mediated ULK1 phosphorylation inhibits the ULK1 function. Thus, the ULK1 kinase activity is activated under autophagy-inducing conditions. ULK1 further phosphorylates and activates the class III VPS34-BECN1 phosphatidylinositol 3-kinase (PI3KC3) complex, which produces phosphatidylinositol 3-phosphate (Ptdlns3P) on the isolation membrane, the nascent forming autophagosomal membrane. BECN1 is the mammalian homolog of yeast Atg6, and stabilizes and activates the VPS34 kinase complex. Ptdlns3P is important for isolation membrane growth through recruiting WD repeat domain phosphoinositide-interacting (WIPI/ATG18) proteins and ATG9-containing vesicles. Among all ATG proteins, ATG9 is the only integral membrane protein essential for autophagosome formation and functions as a lipid scramblase (Matoba et al., 2020; Ghanbarpour et al., 2021). At the steady state, ATG9 is localized at the trans-Golgi network and recycling endosomes, and upon autophagy induction, ATG9-containing vesicles are translocated to the autophagosome assembly site to drive the expansion of the isolation membrane. The transport of ATG9 is regulated by the late endosome-associated small GTPase RAB7.

On the isolation membrane, yeast Atg8, and its mammalian homologs including LC3 (microtubule-associated protein light chain 3) and GABARAPs (GABA Type A receptor-associated proteins), are conjugated to the lipid phosphatidylethanolamine (PE) through a multi-step ubiquitination-like conjugation process regulated by ATG4, ATG7, ATG3 and the ATG12-ATG5-ATG16L1 complex. Isolation membrane-localized LC3 is essential for autophagosome formation, as well as for cargo recruitment via its binding to autophagy cargo receptors. Completed autophagosomes fuse with the lysosome and form autolysosomes, where autophagic substrates are degraded. Multiple protein complexes involved in membrane fusion participate in the autophagosome-lysosome fusion step, including the HOPS (homotypic fusion and protein sorting) complex, the SNARE complex, and RAB proteins.

## Removal of Damaged or Excess Cellular Structures

### Overall Roles of Autophagy in Metabolic Regulation

Whole-body knockout (KO) of several essential ATG genes, including *Atg5*, *Becn1* and *Atg7*, leads to embryonic or postnatal lethality, suggesting that autophagy is essential for embryonic and postnatal development. Thus, studies on energy metabolism using whole-body ATG gene KO mice are rare and are based on KO mouse models with partial autophagy reduction, such as *Atg4b*-null mice and *Becn1* heterozygous mice. ATG4 family proteins function in LC3/GABARAP processing and delipidation, and are composed of four isoforms, ATG4A, ATG4B, ATG4C and ATG4D, among which ATG4B possesses the highest processing activity (Maruyama and Noda, 2018). *Atg4b*-null mice show partial autophagy inhibition and do not show embryonic lethality phenotypes. In response to high-fat diet feeding, *Atg4b*-null mice have hepatic steatosis, decreased energy expenditure, increased weight gain and disrupted blood glucose clearance, compared to wild-type mice (Fernández et al., 2017). Similarly, *Becn1* heterozygous mice also show partially decreased autophagy activity in metabolic organs, and systemic glucose intolerance and insulin intolerance upon high-fat diet feeding, even though their body weight is comparable to that of wild-type mice (Yamamoto et al., 2018). These studies suggest that systemic suppression of autophagy activity disrupts energy homeostasis and exacerbates metabolic dysfunctions.

On the other hand, activating autophagy has different metabolic effects among various metabolic organs, since the demands of autophagy activity in each metabolic organ are different. Ubiquitous *Atg5*-overexpressing transgenic mice show improved systemic glucose clearance, decreased fat and body mass upon aging, and extended lifespan compared to wild-type mice (Pyo et al., 2013). These beneficial effects are attributed to increased autophagy activity and antioxidant levels via Atg5 overexpression. In line with this, mutant mice defective in stress-induced autophagy but not basal autophagy, BCL2^AAA^ mice, fail to show improvements in glucose metabolism induced by exercise (He et al., 2012), suggesting that whole-body autophagy activation during exercise is metabolically beneficial and essential for exercise-induced metabolic improvements. Furthermore, pharmacological activation of autophagy also shows overall beneficial effects on energy metabolism homeostasis. For example, spermidine and Rg2 activate bulk autophagy through inhibition of the histone acetyltransferase EP300, and activation of the AMPK-ULK1 signaling pathway, respectively (Pietrocola et al., 2015; Fan et al., 2017). Short-term *in vivo* administration of these autophagy inducers suppresses lipid accumulation in the white adipose tissue and liver, and limits body weight gain in response to high-fat diet treatment, resulting in improved glucose tolerance and insulin sensitivity (Fan et al., 2017; Fernández et al., 2017). These findings highlight that autophagy activation is important to maintain whole-body energy homeostasis. However, long-term genetic or pharmacological activation of autophagy may also negatively impact metabolism. Although hyperactivating autophagy by a constitutively active BECN1 mutant (BECN1^F121A^) suppresses ER stress and improves systemic insulin sensitivity in response to high-fat diet feeding, it reduces insulin storage in pancreatic β cells and causes systemic glucose intolerance by excessive degradation of insulin granules, which can be reversed by treatment of autophagy inhibitors (Yamamoto et al., 2018). Consistently, in response to high-fat diet treatment, partial suppression of the VPS34 kinase by systemic heterozygous expression of a kinase-dead VPS34 mutant (Vps34^D761A/+^), as well as VPS34 inhibitor treatment, suppresses hepatic steatosis and enhances glucose tolerance and insulin sensitivity, via suppression of metabolic substrate supplies to mitochondria, activation of AMPK signaling pathway, and increase of fatty acid β-oxidation (Bilanges et al., 2017). Thus, the roles of autophagy in energy metabolism are complex, and further investigations are necessary to discover tissue-specific mechanisms of ATG proteins in metabolic regulation.

### Lipophagy

Lipid droplets are ubiquitous lipid-storing organelles, composed of a core of neutral lipids (such as triacylglycerol) surrounded by a phospholipid monolayer. Lipid droplets play a pivotal role in lipid energy metabolism, and also in gene expression by providing the ligands for peroxisome proliferator-activated receptors (PPARs). The canonical lipid droplet degradation pathway, lipolysis, occurs in the cytosol and is controlled by cytosolic lipases, including adipose triglyceride lipase (ATGL) and hormone-sensitive lipase (HSL). Lipolysis stimuli, such as norepinephrine stimulation, induce phosphorylation of cytosolic lipases and lipid droplet-associated proteins (such as PLINs) through the PKA signaling pathway, resulting in increased lipolysis. In the past decade, growing evidence has revealed that macroautophagy also functions as a lipid droplet degradation pathway, known as lipophagy (Kounakis et al., 2019) (Figure 1). Through lipophagy, triacylglycerol and cholesterol esters stored in lipid droplets are delivered to the lysosomes for degradation and lipids are released for reuse. The direct evidence is the presence of accumulated lipid droplets within double-membrane autophagosomal structures. Pharmacological or genetic (knockdown of Atg5) inhibition of autophagy in cultured hepatocytes increases intracellular lipid contents. Consistently, liver-specific KO of Atg7 in mice increases hepatic lipid levels compared with those of control mice (Singh et al., 2009a), whereas liver-specific KO of Rubicon, an autophagy-inhibiting protein, ameliorates hepatic lipid accumulation in response to high-fat diet challenge (Tanaka et al., 2016). Moreover, RAB proteins are also shown to participate in lipophagy (Schroeder et al., 2015; Li et al., 2016). However, although many efforts have been devoted to lipophagy research, the molecular mechanism of lipophagy is still largely unclear. Notably, lipophagy-specific receptors have not been identified until recently. Spartin, a protein mutated in the Troyer syndrome characterized by the degeneration of upper motor neurons (Patel et al., 2002), is identified as a lipophagy receptor (Chung et al., 2021). Spartin localizes to lipid droplets and mediates autophagy-dependent lipid droplet delivery to lysosomes through interacting with LC3A and LC3C, although a canonical LC3-interacting region (LIR) motif has not been determined. KO of Spartin, or expression of the Spartin dominant-negative mutant, leads to accumulation of lipid droplets in breast cancer cells and mouse motor cortex neurons. These findings support that the macroautophagy machinery regulates cellular lipid turnover. Furthermore, a positive interplay between lipophagy and lipolysis has been discovered. In brown adipose tissue, autophagosomes and autophagosome-associated LC3 act as scaffolds for cytosolic lipases, such as ATGL, to localize on lipid droplets, where lipophagy and cytosolic lipolysis complement each other in lipid droplet degradation (Martinez-Lopez et al., 2016). Future investigation is needed to further demonstrate the molecular mechanisms and the physiological functions of lipophagy.

In addition, CMA also facilitates lipid droplet degradation. Although it does not directly break down lipid droplets, CMA degrades the lipid droplet-associated coating proteins, PLIN2 and PLIN3, which contain the KFERQ targeting motif. CMA-mediated PLIN degradation enhances the translocation of both cytosolic lipases and macroautophagic proteins onto the lipid droplets for degradation (Kaushik and Cuervo, 2015; 2016).

### Mitophagy

Mitochondria are the main site of energy production and the powerhouse of the cell, generating up to 95% of ATP in the cell. Under nutrient-rich conditions, mitochondria generate reactive oxygen species, which in turn induce mitochondria malfunction and lead to systemic energy disruption. Mitophagy is an important mitochondrial quality control pathway that selectively eliminates dysfunctional mitochondria (Palikaras et al., 2018) (Figure 1). Several mitophagy-mediated proteins are identified, including PTEN-induced kinase 1 (PINK1) and the cytosolic E3 ubiquitin ligase Parkin, and the mitochondrial outer-membrane proteins BNIP3 and FUNDC1. PINK1 accumulates specifically on the surface of damaged mitochondria by defective proteolytic cleavage due to the loss of mitochondrial membrane potential. Accumulated PINK1 recruits, phosphorylates and activates Parkin on the mitochondrial membrane, and active Parkin then polyubiquitinates outer mitochondrial membrane proteins. Ubiquitinated damaged mitochondria are recognized and bound by mitophagy receptors, such as optineurin and NDP52 (Lazarou et al., 2015), through the interaction between mitophagy receptors and LC3 via the LC3-binding LIR motif, leading to mitophagy induction.

However, the metabolic role of mitophagy is not fully understood. KO of either *Bnip3* or *Fundc1* specifically in the liver or adipose tissue leads to damaged mitochondria accumulation, excess lipid deposition, and systemic insulin resistance and obesity (Glick et al., 2012; Wu et al., 2019). However, by contrast, although skeletal muscle-specific *Fundc1* KO mice also show accumulation of damaged mitochondria and increased levels of reactive oxygen species in skeletal muscle as whole-body and adipose tissue-specific *Fundc1* KO mice (Fu et al., 2018), they have ameliorated insulin resistance, hepatic steatosis and obesity in response to high-fat diet challenge, due to enhanced secretion of FGF21 from muscle, a stress-induced metabolically beneficial myokine (muscle-secreted hormone). In line with this, skeletal muscle-specific *Atg7* deletion also induces mitochondrial dysfunction and increases Atf4-dependent FGF21 secretion, resulting in increased fatty acid β-oxidation, browning of white adipose tissue, and systemic energy metabolism improvement in response to high-fat diet treatment (Kim et al., 2013). Thus, FUNDC1, or FUNDC1 and ATG7-mediated mitophagy, may have different overall effects on systemic metabolism in different metabolic tissues via both cell autonomous and non-cell autonomous mechanisms.

Notably, physical exercise is a potent autophagy inducer in skeletal muscle (Rocchi and He, 2017). During exercise, mitochondrial oxidative stress in the contracting muscle is significantly increased, leading to activation of mitophagy (Laker et al., 2017). Exercise-induced mitophagy plays an important role in both mitochondrial quality control in the muscle and exercise training-induced metabolic adaptation. In response to energetic stress induced by acute exercise, specific isoforms of AMPKs (AMPKα1, α2, β2 and γ1) are found to be localized and activated on the mitochondrial outer membrane (Drake et al., 2021). Activated AMPK then phosphorylates and activates ULK1, which activates the downstream autophagy machinery and mitophagy. In line with this, KO of ULK1 specifically in the muscle abolishes the metabolic adaptations to exercise (Laker et al., 2017).

### ER-phagy

The endoplasmic reticulum (ER) plays pivotal roles in protein synthesis and folding, calcium ion storage, and lipid synthesis and lipid droplet formation. The ER is also known as the primary membrane supplier for the isolation membrane. Misfold proteins, saturated fatty acids, and reactive oxygen spices induce ER stress, which impairs ER function and cellular homeostasis and is implicated in obesity and diabetes progression. There are two major ways of ER quality control, one is the unfolded protein response (UPR), and the other is ER-phagy (specific degradation of the ER by macroautophagy) (Figure 1). UPR is triggered by ER stress and activates several transcriptional factors including ATF6, ATF4, CHOP and XBP1, which upregulate downstream genes encoding chaperones and ER-associated degradation proteins to eliminate causes of ER stress (Yang et al., 2021). Notably, these transcriptional factors also upregulate the expression of ATG genes, including BECN1 and ATG7, in mouse embryonic fibroblasts (MEFs) (B'Chir et al., 2013). The ATG proteins participate in ER-phagy to regulate ER homeostasis. *In vivo Atg7* knockdown by adenovirus results in increased ER stress in the liver and disruption of systemic glucose metabolism (Yang et al., 2010). In the liver of obese mice, the levels of key autophagy proteins, such as BECN1, ATG7 and ATG5, are decreased. Conversely, autophagy activation by overexpression of Atg7 or knock-in of the hyperactive mutant BECN1^F121A^ reduces ER stress and improves both liver energy homeostasis and systemic insulin sensitivity (Yang et al., 2010; Yamamoto et al., 2018). Consistent with these studies, systemic administration of the autophagy activator rapamycin to Akita mice, a type 1 diabetes model with misfolded proinsulin accumulation, ameliorates diabetic phenotypes through activating autophagy and reducing ER stress and β cell apoptosis (Bachar-Wikstrom et al., 2013).

So far, eight ER-phagy receptors have been identified, FAM134 A/B/C, SEC62, RTN3, CCPG1, TEX264 and ATL3 (Khaminets et al., 2015; Fumagalli et al., 2016; Grumati et al., 2017; Smith et al., 2018; Chen et al., 2019; Chino et al., 2019). All the identified ER-phagy receptors are ER membrane proteins and are able to interact with LC3/GABARAP through the LIR motif. Among them, FAM134B is the best characterized ER-phagy receptor. ER stress induces FAM134B phosphorylation through activated CAMK2B, which enhances FAM134B oligomerization. Activation of FAM134B leads to enhanced fragmentation of ER membranes, followed by subsequent degradation via ER-phagy (Jiang et al., 2020). The other two FAM134 family proteins, FAM134A and FAM134C, also function as ER-phagy receptors, but there are a number of differences among the three. FAM134B is functional under both basal and stress conditions (Khaminets et al., 2015), whereas FAM134A and FAM134C are inactive under basal conditions and only activated by stress stimuli, and thus are key for stress-induced ER fragmentation and autophagic degradation (Kumar et al., 2021; Reggio et al., 2021). Furthermore, although all three FAM134 proteins possess the LIR domain, for pro-collagen I homeostasis, FAM134A functions also in a LIR-independent manner and compensates for the loss of FAM134B or FAM134C. In comparison, FAM134C is unable to compensate for the loss of other FAM134 proteins and functions coordinately with FAM134B (Reggio et al., 2021).

Specifically, ER-accumulated aggregates of prohormones, such as *Akita* mutant proinsulin, are eliminated via RTN3-dependent ER-phagy (Cunningham et al., 2019). In *Akita* mice, the C96Y mutation in *Insulin 2* (*Ins2* ^C96Y^) blocks protein folding and induces proinsulin aggregation in the ER, resulting in type 1 diabetes progression. These insoluble aggregates are unable to be removed by the ER luminal chaperon glucose-regulated protein 170 (GRP170); instead, they are cleared by RTN3-mediated ER-phagy. The RTN3-dependent ER-phagy pathway is also involved in the elimination of other misfolded or aggregated prohormones, including pro-opiomelanocortin (POMC) and pro-arginine-vasopressin (Pro-AVP) (Cunningham et al., 2019). Prohormone aggregates are captured by the transmembrane ER protein progesterone receptor membrane component 1 (PGRMC1) via its luminal domain, and delivered to the RTN3-mediated ER-phagy pathway for clearance (Chen et al., 2021).

In addition, a genome-wide CRISPRi screening reveals that mitochondrial oxidative phosphorylation proteins are important for ER-phagy (Liang et al., 2020), suggesting that different from bulk autophagy, ER-phagy requires normal mitochondrial metabolism and function for execution. Despite the link between ER stress and diabetes, how ER-phagy regulates energy metabolism and metabolic disease progression is largely unknown. Further studies are needed to demonstrate both the cell autonomous and the non-cell autonomous roles of ER-phagy and ER-phagy-related proteins in energy metabolism.

## Regulation of Tissue Development and Homeostasis

### Adipose Tissue Development and Function

White adipocytes are occupied by lipid droplets and are the main storage of body neutral lipids, such as triacylglycerol. In addition to functioning as a fat storage organ, adipose tissue has also been recognized as an endocrine organ in the past 2 decades. Adipose tissues regulate systemic energy metabolism through secreting various adipokines (adipose-derived hormones), such as adiponectin, leptin and resistin. Both *in vitro* and *in vivo* evidence demonstrate that autophagy is required for adipogenesis (Baerga et al., 2009; Singh et al., 2009b; Zhang et al., 2009). In MEFs and 3T3-L1 fibroblast cells, genetic (Atg7 or Atg5 knockdown) or pharmacological (3-methyladenin or chloroquine treatment) inhibition of autophagy blocks adipocyte differentiation, via either promoting apoptosis, suppressing expression of the adipogenesis master regulator PPARγ, or enhancing PPARγ degradation via the ubiquitin-proteasome system (Baerga et al., 2009; Singh et al., 2009b; Zhang et al., 2013). *Atg7* deficiency at early development stages of white adipose tissue increases the mitochondrial content, the fatty acid β-oxidation rate and the number of multilocular lipid droplets, resulting in browning of the white adipocytes. As a result, adipocyte-specific Atg7 KO mice show decreased body weight and white adipose tissue mass, and increased systemic insulin sensitivity (Singh et al., 2009b; Zhang et al., 2009). Similarly, deletion of *Atg7* in brown adipose tissue also causes an increase in mitochondrial contents and β-oxidation, leading to elevated brown adipose tissue mass (Kim et al., 2019). Although the impact of *Atg7* deletion in mature adipocytes remains to be determined, other studies have shown that autophagy also plays an important role in the regulation of post-developmental functions and homeostasis of mature adipocytes. Different from KO of Atg7 during adipose development stages, adipocyte-specific KO of *Becn1* during adulthood increases adipose tissue inflammation, ER stress and apoptosis, and decreases insulin sensitivity (Jin et al., 2021). Similarly, post-developmental adipose-specific deletion of *Atg3* or *Atg16L1* leads to dysfunctional mitochondria accumulation followed by increased lipid peroxides in adipose tissue, although fat mass and body weight appear to be unaffected. Increased lipid peroxides enter the circulation and affect liver energy homeostasis, resulting in systemic insulin resistance (Cai et al., 2018).

In comparison to white adipose tissue, brown adipose tissue functions as a thermogenic organ through non-shivering thermogenesis, and is characterized by high mitochondria contents, UCP1 (uncoupling protein 1) expression levels and fatty acid β-oxidation rates. There are also beige adipocytes, which are brown adipocyte-like cells present in white adipose tissue induced by browning of white adipocytes. Similar to brown adipocytes, beige adipocytes have multilocular lipid droplets, increased mitochondrial contents and upregulated β-oxidation. Activation of adipocyte browning is beneficial for systemic metabolism and potentially a therapeutic target in metabolic disorders, while beige-to-white transition has opposite effects. During beige-to-white adipocyte transition, autophagy is activated and is involved in mitochondrial clearance (Altshuler-Keylin et al., 2016). Accordingly, pharmacological or genetic inhibition of autophagy by chloroquine treatment, or *Atg5*-or *Atg12*-deletion, prevents beige-to-white adipocyte transition and protects against diet-induced obesity and systemic insulin resistance (Altshuler-Keylin et al., 2016).

### β Cell Maintenance

Pancreatic β cells regulate systemic glucose metabolism by sensing circulating glucose and secreting insulin into the bloodstream. Under steady-state conditions, the autophagy activity in β cells is low, and autophagosome formation is upregulated in high-fat diet-induced and genetic mouse models of obesity and diabetes (Ebato et al., 2008; Chu et al., 2015; Sheng et al., 2017). Treatment of free fatty acids in cultured β cells effectively induces autophagy via the JNK1 signaling pathway (Komiya et al., 2010), indirectly explaining the *in vivo* data. β cell-specific *Atg7* deletion decreases β cell mass, intracellular insulin contents and glucose-stimulated insulin secretion, due to accumulated defective organelles, elevated ubiquitinated protein aggregates and increased apoptosis (Ebato et al., 2008; Jung et al., 2008). *Atg7* deletion in β cells results in hyperglycemia and impaired glucose tolerance in both regular and high-fat diet-fed mice. Thus, the basal autophagy activity in β cells plays a critical role in maintaining β cell function and systemic glucose homeostasis.

Yet too much autophagy in β cells can be metabolically detrimental. Although pharmacological activation of autophagy by rapamycin injections in Akita mice improves glucose metabolism, potentially via decreased β cell ER stress and apoptosis (Bachar-Wikstrom et al., 2013), chronic autophagy activation reduces insulin storage in β cells. Insulin granules are an unusual autophagy cargo via autophagic degradation of secretory vesicles (vesicophagy) (Figure 1). Chronic constitutive activation of autophagy by BECN1^F121A^ leads to excessive degradation of insulin granules in β cells, resulting in decreased insulin storage and secretion and impaired systemic glucose tolerance in response to high-fat diet feeding, even though systemic insulin sensitivity is better than wild-type mice (Yamamoto et al., 2018). The vesicophagy degradation process is different from crinophagy, with the latter representing the direct fusion of secretory vesicles with lysosomes. Although the key autophagy proteins ATG5, BECN1 and ATG7 are potentially involved in vesicophagy, the molecular mechanism is largely unknown and needs further investigation. Furthermore, due to their insulin-secreting function, pancreatic β cells are more sensitive to autophagy level changes than other metabolic organs and cells; accordingly, the autophagy activity needs to be tightly regulated in β cells to maintain their homeostasis and function.

### Liver Lipid Metabolism

The liver is an important metabolic organ regulating systemic energy homeostasis through gluconeogenesis and lipogenesis. In the liver, in addition to bulk autophagy, various selective autophagy pathways, including mitophagy and lipophagy, regulate liver energy homeostasis (Ueno and Komatsu, 2017). Overnutrition causes excess ectopic lipid accumulation in the liver, which has a causal link to the pathogenesis of non-alcoholic fatty liver disease (NAFLD), steatohepatitis (NASH) and hepatocellular cancer. In the liver of obese mice, the expression of several key ATG proteins, including ATG5, BECN1 and ATG7, is decreased, suppressing the autophagy flux (Yang et al., 2010). Pharmacological and genetic activation of autophagy by rapamycin treatment or *Atg7* overexpression in the liver decreases lipid accumulation, alleviates ER stress and improves insulin action, resulting in enhanced systemic glucose homeostasis. Conversely, KO of ATG genes, including *Atg7*, *FIP200* and the PI3KC3 *Vps34*, leads to accumulation of ubiquitin-positive aggregates and deformed organelles in the liver. These KO mice show hepatomegaly, hepatic steatosis, and exacerbated systemic glucose intolerance and insulin resistance (Komatsu et al., 2005; Yang et al., 2010; Jaber et al., 2012; Ma et al., 2013). Notably, many of these ATG gene KO mice have reduced glycogen contents but increased lipid storage levels in the liver, raising the possibility that hepatic autophagy deficiency may induce a shift of energy substrates from lipids to carbohydrates through altering gene expression. Indeed, autophagy is reported to regulate metabolic gene expression in the liver by degrading CRY1, an inhibitor of master circadian rhythm transcription factors CLOCK and BMAL1, via several LC3-interacting LIR motifs in CRY1 (Toledo et al., 2018). The expression of many energy metabolic genes is regulated by the circadian rhythm. Autophagy deficiency leads to hepatic accumulation of CRY1, but not other circadian rhythm-related proteins such as PER1, BMAL1 and CLOCK, resulting in the disruption of the circadian rhythm and nutrient metabolism.

## Cancer Metabolism in Metabolic Tissues

Metabolic organs, including liver and pancreas, develop a variety of malignancies. Adipose tissues and muscles rarely develop cancer likely due to their largely quiescent status. Depending on tumor types, stages and genetic context, autophagy acts as a double-edged sword and has both tumor-suppressing and oncogenic roles in cancer development. Neoplasia has been found in the liver and pancreas of various autophagy-deficient mouse models, including *Becn1* heterozygous mice (Qu et al., 2003; Yue et al., 2003), systemic mosaic *Atg5* deletion mice (Takamura et al., 2011), and liver- or pancreas-specific *Atg5* or *Atg7* deletion mice (Inami et al., 2011; Takamura et al., 2011; Rosenfeldt et al., 2013; Yang et al., 2014). Autophagy suppresses tumor initiation by maintaining cellular homeostasis through quality control of proteins, organelles and chromosomal stability (Mathew et al., 2007). However, lesions in autophagy-deficient mice are benign or premalignant, and do not progress to high-grade malignancy (Qu et al., 2003; Takamura et al., 2011; Rosenfeldt et al., 2013). Rather, in established tumors, genetic or pharmacological inhibition of autophagy suppresses pancreatic tumor growth (Yang et al., 2011; Yang et al., 2014; Yang et al., 2018). The tumor-promoting role of autophagy can be explained by two mechanisms. First, in established cancer cells, inhibition of autophagy causes the accumulation of defective mitochondria, which leads to a reduction in oxidative phosphorylation and suppression in tumor growth (Guo et al., 2011; Yang et al., 2011). Second, the autophagy-lysosomal pathway, regulated by the MiT/TFE family of transcription factors, plays an important role in maintaining cellular amino acid pools, which is essential for pancreatic cancer cell growth and proliferation under stress conditions (Perera et al., 2015). Thus, autophagy plays opposing roles in the initiation versus progression of tumors.

Tumors rely on nutrient supplies from the host. Autophagy is also important for tumor growth by regulating host energy metabolism and tumor environment (Kimmelman and White, 2017; Poillet-Perez and White, 2019). There are two main nutrient supply routes for tumors: one is the local tumor microenvironment, and the other is the host circulation. In the pancreatic tumor microenvironment, stroma-associated pancreatic stellate cells (PSCs) secrete alanine, a non-essential amino acid, through the autophagic pathway activated by signals released from cancer cells (Sousa et al., 2016). PSC-derived alanine serves as an alternative carbon source of pancreatic cancer cells. Host autophagy also maintains tumor growth by sustaining the level of circulating arginine, a semi-essential amino acid. In whole-body *Atg7* or *Atg5* KO mice, circulating arginine levels are decreased, resulting in suppressed tumor growth (Poillet-Perez et al., 2018). Mechanistically, liver damage and steatosis caused by autophagy deficiency in these mice promotes the release of arginase 1 (ARG1) into the bloodstream, which catalyzes the degradation of arginine into ornithine and subsequently decreases circulating arginine levels.

## Non-Degradative Functions of Autophagy

### Conventional and Unconventional Secretion

**FIGURE 2:**
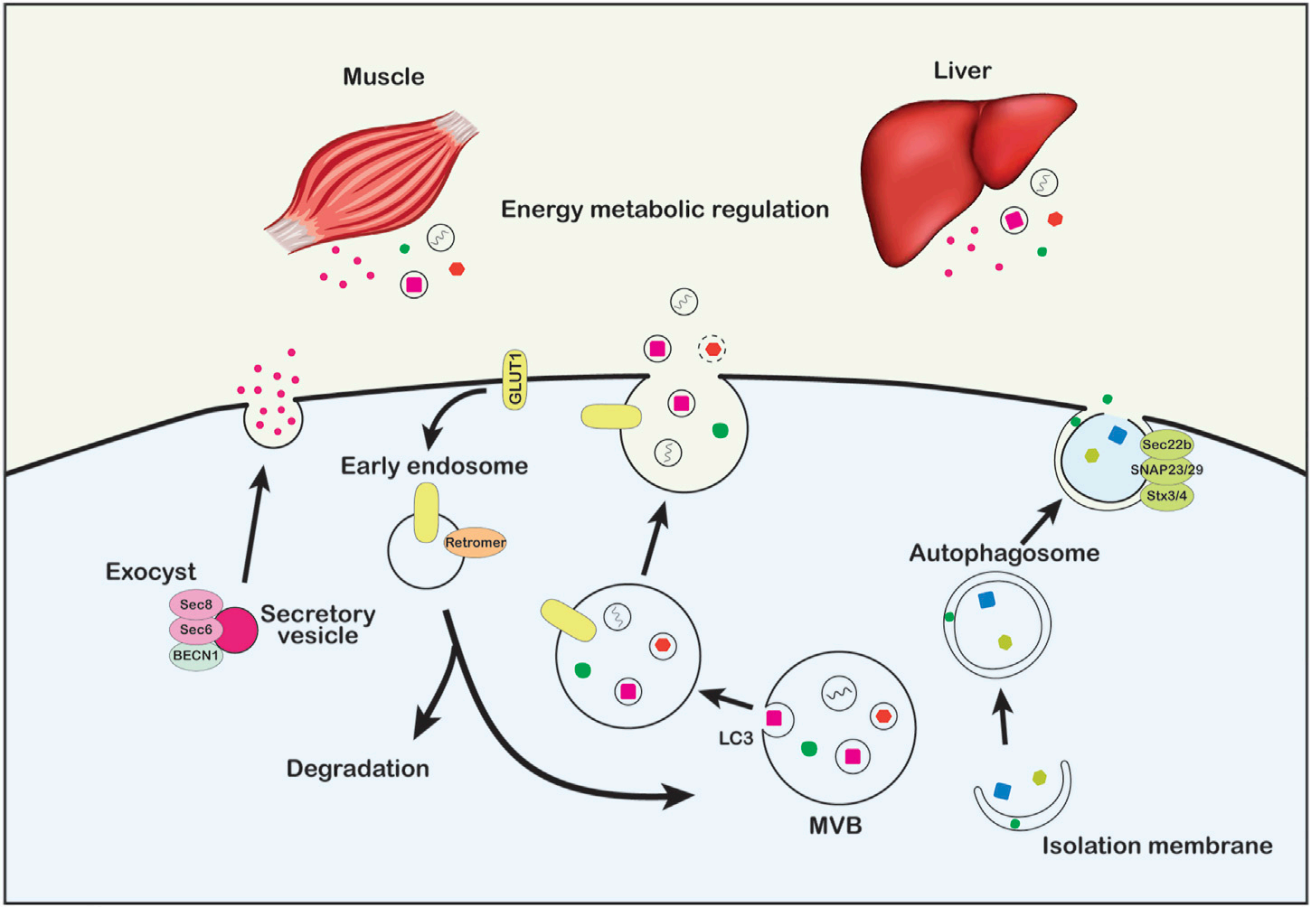
Non-canonical functions of the autophagy machinery in vesicle trafficking and secretion. ATG proteins regulate systemic metabolic and energy balance via non-degradative functions in vesicle trafficking and secretory protein release, including adiponectin secretion through direct interaction between BECN1 and the exocyst protein (left), retromer-driven GLUT1 recycling and ATG5- and LC3-dependent exosome secretion through the endosomal and MVB pathway (middle), and secretory autophagy (right).

Besides degradative functions, autophagy proteins promote protein secretion from both conventional and unconventional secretory pathways (Figure 2). The autophagy protein BECN1 facilitates the secretion of adiponectin, an insulin-sensitizing adipokine, independent of the canonical degradation function. In adipocytes, BECN1 interacts with several components of the exocyst complex (including SEC5, SEC6 and SEC8), a conserved octameric complex tethering secretory vesicles to the plasma membrane. The hyperactive BECN1^F121A^ mutant shows stronger binding to the exocyst components, leading to increased adiponectin secretion from adipocytes and improved systemic insulin sensitivity and energy homeostasis, via activation of AMPK signaling in metabolic organs by adiponectin (Kuramoto et al., 2021). Notably, different from adiponectin vesicles, vesicles containing several other adipokines, including leptin and resistin, are not efficiently recruited to the BECN1-exocyst complex, and their secretion is not regulated by BECN1. Thus, various types of secretory vesicles may occupy different subcellular locations and are sorted by distinct pathways from the trans-Golgi network to the plasma membrane. Further investigation is needed to reveal how BECN1 distinguishes adiponectin vesicles from other types of secretory vesicles. In addition, besides regulating hormonal secretion, the exocyst is also involved in the plasma membrane translocation of cell surface receptors such as the glucose transporter type 4 (GLUT4), which uptakes glucose in an insulin-responsive manner (Wang et al., 2019). Thus, it is possible that the autophagy machinery also plays a role in exocyst-dependent protein trafficking to the cell surface.

Most secretory proteins possess a canonical N-terminal signal peptide, which regulates secretory protein transport to the ER and the Golgi apparatus, and protein secretion via exocytosis. This pathway is known as the conventional secretory pathway. Over the past decade, a growing body of evidence shows that certain cytosolic proteins lacking the signal peptide, known as leaderless cargos, are secreted through unconventional secretory pathways, bypassing the ER-Golgi secretory pathway. There are four main types of unconventional secretory pathways: Type I is pore-mediated secretion; Type II is ABC transporter-mediated secretion; Type III is endocytic compartment-mediated secretion; and Type IV is Golgi-bypassed secretion (Rabouille, 2017; Padmanabhan and Manjithaya, 2020). Several ATG proteins, such as ATG5, ATG16L1, BECN1 and LC3, are involved in the Type III unconventional secretory pathway, termed secretory autophagy (Figure 2). A number of leaderless cargo proteins of secretory autophagy have been identified, including IL-1β and FABP4 (fatty acid binding protein 4). In response to lysosomal damage, IL-1β is reported to translocate into the intermembrane space or inner lumen of LC3-positive autophagosome-like vesicles (Dupont et al., 2011). These LC3-positive vesicles escape from Syntaxin (Stx) 17-dependent autophagosome-lysosome fusion, and fuse with the plasma membrane through the Sec22b-SNAP23/29-Stx3/4 complex for IL-1β secretion (Kimura et al., 2017). In Atg5 KO bone marrow-derived macrophages, IL-1β secretion is decreased compared with wild-type macrophages (Dupont et al., 2011).

Recent studies show that FABP4 is also secreted by the unconventional secretory autophagy. FABP4, also known as adipocyte protein 2 (aP2), is highly expressed in adipocytes and secreted as an adipokine. FABP4 functions as a lipid chaperone protein, and plays a key role in intracellular fatty acid metabolism, hepatic glucose production and insulin secretion (Prentice et al., 2019). FABP4 secretion from white adipocytes requires early components of the autophagy pathway such as FIP200, ULK1 and BECN1, but not ATG5 (Josephrajan et al., 2019). The molecular mechanism of FABP4 secretion through the autophagic secretory pathway is not fully elucidated, but it appears that it is different from ATG5-and LC3-dependent IL-1β secretion.

Recently, leaderless cargos have also been reported to be translocated into the ER-Golgi intermediate compartment through TMED10, a protein channel, and are then secreted along the secretory pathway (Zhang et al., 2020). TMED10 regulates the secretion of multiple leaderless cargos, including IL-1β, HSPB5, galectin1/3 and Tau. After the chaperone HSP90A unfolds the leaderless cargos in the cytosol, TMED10 binds and trans-locates the cargos into the ER-Golgi intermediate compartment for secretory vesicle packaging (Zhang et al., 2015; Zhang et al., 2020). How TMED10-mediated secretion crosstalks with the autophagy pathway remains unclear.

In addition, many studies have shown that autophagy proteins can indirectly regulate the secretion of neurotransmitters, neuropeptides, cytokines and secretory enzymes via modulating cellular stress responses, receptor metabolism and lipid metabolism (Saitoh et al., 2008; Kaushik et al., 2011; Bel et al., 2017; Kim et al., 2021). Thus, autophagy plays versatile roles in metabolism not only cell autonomously by degradation, but also non-cell autonomously via protein secretion.

### Cell-Surface Receptor Recycling

The stability of cell-surface proteins, such as receptors and transporters, is important in maintaining cellular functions. The endosome-Golgi retrieval pathway plays a critical role in regulating the level of plasma membrane proteins, determining whether they are recycled to the plasma membrane or transported to the lysosome for degradation. The retromer is the key membrane protein sorting complex at the endosome, composed of Vacuolar Protein Sorting (VPS) 26, VPS29 and VPS35 (Chandra et al., 2021). The retromer sorts membrane proteins to the plasma membrane or trans-Golgi network, and prevents their degradation by lysosomes. The cell-surface level of glucose transporter 1 (GLUT1), also known as Solute Carrier Family 2 Member 1 (SLCA1) for glucose uptake, is regulated by the autophagy machinery via TBC1D5, a retromer-associated GTPase-activating protein specifically for RAB7 (Jimenez-Orgaz et al., 2018) (Figure 2). Inhibition of TBC1D5 activates RAB7, and promotes membrane association of the retromer and retromer-mediated receptor recycling. Under metabolic conditions relying on glycolysis, such as Ras transformation, hypoxia or glucose starvation, TBC1D5 binds with LC3 through a canonical LIR motif and re-localizes to LC3-positive autophagosomes from the retromer complex. This leads to retromer activation, increased GLUT1 recycling to the plasma membrane, and increased glucose uptake in autophagy-competent cells, but not in Atg5 or Atg7 KO cells (Roy et al., 2017). These findings suggest that GLUT1 recycling is dependent on the autophagy pathway. Given GLUT1 is ubiquitously expressed and its level is increased in many types of cancer cells, targeting autophagy may limit glucose metabolism in cancer cells and have therapeutic potential against tumors (Ancey et al., 2018). Notably, even though TBC1D5 is incorporated inside autophagosomes, the total TBC1D5 protein level is unchanged under the glucose starvation condition. Further investigation is needed to reveal the mechanism by which TBC1D5 escapes from autophagic degradation.

### Exosome Secretion

The autophagy machinery has recently been reported to regulate the secretion of extracellular vesicles and exosomes (Figure 2). Exosomes are small extracellular vesicles with a diameter of 50–150 nm, containing various types of cargos including microRNAs, lipids, enzymes and cytokines. Exosomes regulate a variety of physiological processes, such as tumor metastasis and energy metabolism, through cell-to-cell signaling (Guay and Regazzi, 2017). Exosomes are derived from invaginated vesicles inside multivesicular bodies (MVBs) in the endosomal pathway. MVBs face two fates: one is degradation via fusion with the lysosome, and the other is secretion as exosomes by translocation to the plasma membrane. Several key autophagy proteins, including ATG5, ATG16L1 and LC3, regulate the fate determination of MVBs. LC3 interacts with ATPase H^+^ Transporting V1 Subunit E1 (ATP6V1E1), a component of the vacuolar ATPase (V-ATPase) involved in acidification of MVBs. LC3 and ATP6V1E1 are internalized into MVBs through an ATG5-dependent manner, resulting in increased MVB pH and exosome secretion (Guo et al., 2017). Certain exosomal cargos, such as RNA-binding proteins, can also interact with LC3 for packaging into exosomes (Leidal et al., 2020). This exosomal secretory mechanism is independent of additional canonical autophagy proteins, besides those required for LC3-PE conjugation. However, the role of the ATG5-and LC3-dependent exosomal secretion in metabolic regulation is unclear. In addition, whether the LC3 conjugation machinery is involved in the sorting of other MVB cargos towards secretion, and how synergism is achieved between classical MVB sorting proteins (such as RAB proteins) and the LC3 conjugation system, also need further investigation.

## Summary and Unanswered Questions

Autophagy-related proteins play complex roles in metabolism, regulating not only the canonical autophagic degradation pathway, but also vesicle trafficking and proteins secretion. Compared to the degradative functions of ATG proteins, their roles in the regulation of cross-talk between metabolic organs via non-canonical, non-degradative, pathways are poorly understood. Autophagy may play more critical roles in endocrine organs than previously thought. For canonical autophagy, although various types of selective autophagy have thus far been discovered, such as mitophagy, lipophagy and ER-phagy, the molecular mechanisms and cargo receptors, which determine the degradation specificity of selective autophagy, remain to be fully elucidated. Furthermore, many single nucleotide polymorphisms (SNPs) on ATG genes have been identified to associate with the onset of metabolic diseases by genome-wide association studies, for example, rs10512488 in *Becn1* (Tamargo-Gómez et al., 2020), but their potential functions and mechanisms in the regulation of autophagy activity and energy metabolism are still unknown. Lastly, because autophagy plays an essential role in organ development and homeostasis, spatiotemporal-specific inhibition or activation of ATG proteins is needed to better understand how autophagy and ATG proteins regulate nutrient and energy homeostasis.
